# A Bayesian Decision Theory Approach for Genomic Selection

**DOI:** 10.1534/g3.118.200430

**Published:** 2018-07-18

**Authors:** Bartolo de Jesús Villar-Hernández, Sergio Pérez-Elizalde, José Crossa, Paulino Pérez-Rodríguez, Fernando H. Toledo, Juan Burgueño

**Affiliations:** * Colegio de Postgraduados, Montecillos, Edo. de México, México; † Biometrics and Statistics Unit, Genetic Resources Program, International Maize and Wheat Improvement Center (CIMMYT), Apdo. Postal 6-641, 06600, México, D.F, México

**Keywords:** Bayesian Decision Theory, Genomic Selection, Loss Function, Simulation Scenarios, GenPred, Shared Data Resources

## Abstract

Plant and animal breeders are interested in selecting the best individuals from a candidate set for the next breeding cycle. In this paper, we propose a formal method under the Bayesian decision theory framework to tackle the selection problem based on genomic selection (GS) in single- and multi-trait settings. We proposed and tested three univariate loss functions (Kullback-Leibler, KL; Continuous Ranked Probability Score, CRPS; Linear-Linear loss, LinLin) and their corresponding multivariate generalizations (Kullback-Leibler, KL; Energy Score, EnergyS; and the Multivariate Asymmetric Loss Function, MALF). We derived and expressed all the loss functions in terms of heritability and tested them on a real wheat dataset for one cycle of selection and in a simulated selection program. The performance of each univariate loss function was compared with the standard method of selection (Std) that does not use loss functions. We compared the performance in terms of the selection response and the decrease in the population’s genetic variance during recurrent breeding cycles. Results suggest that it is possible to obtain better performance in a long-term breeding program using the single-trait scheme by selecting 30% of the best individuals in each cycle but not by selecting 10% of the best individuals. For the multi-trait approach, results show that the population mean for all traits under consideration had positive gains, even though two of the traits were negatively correlated. The corresponding population variances were not statistically different from the different loss function during the 10^th^ selection cycle. Using the loss function should be a useful criterion when selecting the candidates for selection for the next breeding cycle.

The breeding process consists of selecting individuals for crossing, each of which has specific traits of interest. Crossing allows alleles to be exchanged between the parents so that diverse individuals are observed in the progeny of future generations. Conventional phenotypic and pedigree breeding is based on truncated selection of the best performing parents, which are intermated to form the next improved population. Candidates are selected based on the breeding value (BV) and genetic merits of single or multiple traits, and decisions are made based on their phenotypic performance in progeny field trials, greenhouse trials, laboratories, etc. Meanwhile, in GS-assisted breeding, all molecular markers are used to predict the BV of the candidates for selection in a population that has been genotyped but not phenotyped (Meuwissen *et al.*, 2001). The main advantages of GS over phenotypic and pedigree-based selection methods are that GS reduces the cost per cycle and increases time efficiency for variety development by shortening the breeding cycle (Crossa *et al.*, 2017).

In breeding, decisions on which individuals to select and intermate to form the improved population are crucial. For a single trait, the selection differential (S) is the difference between the mean of the selected parents ($\mu_{s}$) and the mean of the base population ($\mu_{1}$), whereas the selection response (*R*) is the difference between the mean of the offspring ($\mu_{2}$) of the selected parents and $\mu_{1}.\;$Therefore, $R=h^{2}S$, where $h^{2}$ is the narrow-sense heritability of the trait of interest (Bos and Caligari 2008). Under $h^{2}∼1,$ the mean of the offspring of the selected parents tends to the mean of the selected parents and, therefore, *R*∼*S*, whereas for small $h^{2}∼0$, the mean of the offspring of the selected parents tends to the mean of the base population and thus *R*<<*S*. Therefore, the selection response enables breeders to estimate the expected selection progress before carrying it out (Costa *et al.*, 2008). When selection is applied to improve the economic value and genetic merits of a crop, plant breeding programs are applied to several traits simultaneously and not just to one trait (Falconer and Mackay 1996).

Plant breeding decisions regarding which individuals to select for the next cycle have associated risks. The decisions will be associated with the losses (or gains) in the response to selection (genetic gains); ideally, we want to maximize the genetic gains with minimum risk. Minimizing the risk and maximizing the genetic gains when selecting the parents may be achieved by different methods. Most of the work for selecting the best parents to cross is by truncation. As a result, the subset of individuals from the base population that will be parents for the next selection cycle follows a truncated distribution. However, another way to select parents for crossing is to optimize a subset of parents using pedigree based on co-ancestry information and additive genetic values (Shepherd and Kinghorn 1998). An approach that seeks to minimize inbreeding and co-ancestry in the context of a quadratic optimization problem was proposed by Wray and Goddard (1994) and Brisbane and Gibson (1995).

Recently, Akdemir and Sánchez (2016) developed a genetic algorithm that optimizes genomic mating between parents under GS; they developed a measure called the risk of a mating plan and used it to minimize a function that combines measures of inbreeding with the risk function. Another decision approach applied to optimize the selection of a set of donor parents for the introgression of alleles to recipient individuals was presented by Han *et al.* (2017); the authors proposed framing this introgression process as an algorithm that can be mathematically formulated and optimized.

As mentioned, truncated selection and optimizing genetic mating systems are decision problems that are made under uncertainty. Another approach for selecting the candidates in a single-trait or multi-trait setting is to assess the cost of that decision using a loss function (LF), which ensures the highest profitability given the user’s preferences, that is, by maximizing R. The LF may take into account, among other factors, the correlation between traits, and the mean and variance of those traits, or it may focus on minimizing the distance between the mean of the (theoretical) parental distribution and the mean of the candidates (that is, maximizing *R*). Decisions made during GS are particularly important because, for a few selection cycles, the only tool available to make selections is based only on the predicted BV of the candidates for selection without observing them in field trials, etc. Genomic prediction computes the BV of the unobserved (genotyped) individuals (testing population) using the phenotypic and genotypic data of their parents, ranks the best predicted BV and selects the top, say, 10%. In a recent study, Blondel *et al.* (2018) proposed ranking lines according to their BV by giving the preference to regression models that assign a high rank to lines with high BV.

In a Bayesian framework, the problem of selecting the best parents is concerned with minimizing the posterior expected loss. Suppose that some unknown parameter indexes the statistical model of the observable trait of interest, and the uncertainty about the parameter is represented by a prior distribution. After the data are observed, the uncertainty about model parameters is formally quantified in the posterior predictive distribution of the BV. However, in phenotypic and genomic selection, decisions are made by regarding just the ranking of the candidates predicted for each trait and disregarding the uncertainty attached to the prediction. Here is where LFs play a role in conventional phenotypic or GS, because the breeder’s decisions should be based on both criteria, the whole posterior predictive distributions of BV and the loss attached to the selection of a parent or a set of parents for upcoming selection cycles.

Several divergences between the distributions of candidates and the parents’ may be used as LF. As previously pointed out, those candidates whose distributions are closer to the theoretical parental distribution will have the lowest loss (higher *R*), and the decision is to advance those lines in a breeding program because they reach the desired mean and keep the genetic variance (high $h^{2}$). For example, the Kullback-Leibler (KL) (Kullback and Leibler 1951) LF measures how two probability distributions diverge, and when the two distributions match, the KL is zero. Thus, given the available phenotypic and genomic information, selecting candidates using this approach should be better than using solely the point estimates of BV because it takes into account all the associated uncertainty, namely, the uncertainty due to the estimation of model parameters and the prediction of the BV. Another LF that could be used when selecting candidates in conventional and GS breeding is the Continuous Ranked Probability Score (CRPS) (Gneiting and Raftery 2007) that reflects the distance between the cumulative distributions of two random variables. LFs, KL and CRPS are measures of the divergence between the distributions of the parents (theoretical) and the candidates, and could be used in both single-trait and multi-trait settings; for instance, the Energy distance or Energy Score (EnergyS) is the multivariate CRPS defined in terms of distributions of random vectors (Székely and Rizzo 2013).

The KL and EnergyS methods are symmetric LFs, given that the penalties due to selection of equidistant distributions located on the right and on the left of the target theoretical distribution are equal. However, in plant breeding, distributions with high density on the right (or left, depending on the trait of interest) of the target distribution [individuals with superior values of the trait(s)] should be less penalized because they are the individuals of interest to the breeder (in case the trait to be selected should increase). Thus, asymmetric LFs should play an important role in plant breeding because those individuals with BV distributed on the right and close to the parents’ distribution should be less penalized. A simple asymmetric LF is the linear-linear (LinLin) function (Berk, 2011), which is linear on both sides of the target distribution but with different slopes. A generalization of the LinLin function in a multivariate setting is the Multivariate Asymmetric LF (MALF) (Komunjer and Owyang 2011), which is a function of the distance between predicted BV of individuals’ and parents’ means and a parameter that controls the degree of asymmetry.

Based on the previous considerations, in this study we introduce uncertainty elements related to the decisions made when selecting parents for GS-assisted breeding by using LFs where the space of decisions (*i.e.*, candidates for selection) is the subset of individuals whose BVs exceed some level of truncation on the base population. The main objective of this study was to show how Bayesian decision theory can be used to select parents for the next breeding cycle by minimizing the expected posterior divergence between the distribution of the candidates and the parental distribution and therefore maximizing the expected response to selection (*R*) given the phenotypic, genotypic and genomic information at hand. The specific objectives of this study are: (1) to present a summary of the Bayesian decision theory framework adapted to the problem of selecting the parents for the next GS selection cycle; (2) to describe various univariate and their multivariate generalizations LF; and (3) to compare the ranking of the top 10% and 30% of the candidate lines selected using single-trait and multi-trait LF *vs.* not using LFs from simulated and real wheat data. The connection between the LFs and the genetic gains is shown when deriving the LFs as functions of response to selection (*R*), selection differential (*S*), intensity of selection (*i*), and heritability ($h^{2}$).

## Materials and Methods

### A decision problem

A decision problem is defined in terms of an outcome space, an action space, and an LF (Dawid 2007). Broadly defined, the goal of decision theory is to help choose among actions whose consequences cannot be completely anticipated, typically because they depend on some future or unknown state of the world; in our framework, the actions are the choices of the offspring that will be the parents in the next breeding cycle, which depend on the unknown BVs to be predicted. Expected loss theory handles this choice by assigning a quantitative loss to each decision, a probability to each state of the world, and then selecting an action that minimizes the expected value of the resulting loss. This idea has proven to be a widely applicable description of rational behavior (Parmigiani and Inoue 2009).

Let A be the action space, and a∈A be an action. No action can be taken without potential losses. The LF is denoted by L(θ,a) and represents the associated penalty when a decision maker takes action a∈A, and the real state of nature is θ∈Θ. In a Bayesian statistical framework, the expected loss is the expectation of the LF with respect to the posterior measure, *i.e.*, $E^{\theta |X}L\left(a,\;\theta \right)=\underset{\text{Θ}}{\int }L\left(\theta ,a\right)\pi \left(\theta |x\right)d\theta .$ When comparing two actions, $a_{1}$ and $a_{2}$, after data X have been observed, the preferred action is the one for which the posterior expected loss is smaller. An $a^{*},\;$the action that minimizes the posterior expected loss is called a Bayes action.

### Loss functions in GS

**Figure 1:**
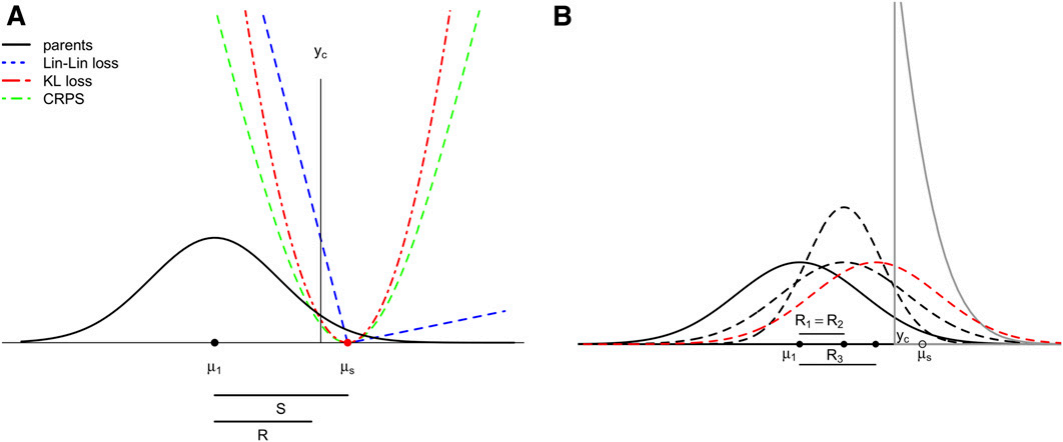
A) Classic idea of selection by truncation at $y_{c}$. Loss functions need to be minimized at $\mu_{s}$ in order to favor lines with high response to selection. Losses were standardized by subtracting the minimum value for representation. Loss functions Kullback-Leibler (KL) and Continuous Ranked Probability Score (CRPS) are symmetric on both sides of target $\mu_{s}$, while LinLin loss is asymmetric. B) The solid line in black represents the base population, the solid gray line corresponds to the truncated distribution after censoring at $y_{c}$ representing the breeder’s preferences. Dashed lines are theoretical distributions of three possible candidates. The candidates’ distribution with mean close to the theoretical $\mu_{s}$ (the greater R red dashed line) and variance similar to that of the parent distribution has the minimum loss.

In our particular case, the action space comprises the set of candidate lines for selection; we expect to assign a loss given our preferences, and then select the best individuals based on the expected loss. To explain the idea of selection under the decision theory approach, we first focused on a single quantitative trait where three LFs are proposed. In addition, we focused on traits whose phenotypic values we wanted to increase in successive selection cycles. Figure 1 illustrates the idea of selection under LFs for a univariate trait; Figure 1a depicts the distribution of the base population that is truncated at $y_{c}$, meaning that the selected individuals will have mean $\mu_{s}$. Parameters $y_{c}$ and $\mu_{s}$ are essential for defining the LF. Figure 1b depicts the three LFs we will describe later; these three functions are minimized when the means of selected candidates is close to $\mu_{s}$, which reflects breeders’ preferences for those individuals with higher response to selection. The gray lines show the theoretical distribution of the trait of interest for individuals according to the breeder’s preference, as well as three possible distributions of candidates. The distribution of the selected candidate is in red because its mean is the closest to $\mu_{s}$ (*i.e.*, higher response to selection) with almost the same variance of the base population; that is, under normality, the divergence between the truncated distribution and the distribution of the selected candidate is the lowest. This idea can be generalized to a multi-trait setting. Below we describe three LFs that we used when selecting candidates to be parents of the next generation.

### Univariate Kullback-Leibler (KL) loss function

The KL divergence reflects how different two probability distributions are. The expected loss of information will be minimal if the two distributions approach each other, and it will be zero if they are identical. Therefore, we can measure how close the distribution of the candidates’ BVs is to the hypothetical parental distribution.

Let Y be a random variable that represents the phenotypic value of a trait of interest in the base population and $Y∼N\left(\mu_{1},\sigma^{2}\right)$; if selection is by truncation (*i.e.*, those realizations of Y falling above a threshold value $y_{c}$ will be the parents of the next breeding cycle), then $Y_{s}$ is the truncated random variable resulting from left truncation at $y_{c}$, which represent the phenotypic values of the selected parents. From properties of normal distribution, $Y_{s}∼N_{T}\left(\mu_{1},\sigma^{2},a=y_{c},b=\infty \right)$, where $N_{T}$ denotes a truncated normal distribution with parameters $\mu_{1},\sigma^{2},a$ and b; but for simplicity, let’s denote $Y_{s}∼N_{T}(\mu_{1},\sigma^{2},y_{c})$. The probability density function of $Y_{s}$ is $\pi \left(y|\mu_{1},\sigma^{2},y_{c}\right)=\frac{1}{z\sigma \sqrt{2\pi }}e^{\frac{-1}{2\sigma^{2}}{\left(y-\mu_{1}\right)}^{2}}$, for $y_{c}\leq y\leq \infty$, and $z=1-\Phi \left(\left(y_{c}-\mu_{1}\right)/\sigma \right)$. Let $Y_{o}$ be a random variable that represents the candidates’ phenotypic values. If we assume normality, then $Y_{o}∼N\left(\mu_{2},\sigma^{2}\right)$. Note that, in order to keep the heritability of the trait, we are assuming that Y and $Y_{o}$ have different means but the same variance. A measure of the divergence between the distributions of $Y_{s}$ and $Y_{o}$ is given by the Kullback-Leibler divergence,

$$D_{KL}\left(F_{Y_{o}},F_{Y_{s}}\right)=\overset{\infty }{\underset{y_{c}}{\int }}\log\frac{N_{T}(\mu_{1},\sigma^{2},y_{c})}{N(\mu_{2},\sigma^{2})}N_{T}(\mu_{1},\sigma^{2},y_{c})dy,$$ (1)

which we want to minimize in the breeding selection framework.

After evaluating the integral in (1) and simplifying (see Appendix A), the KL loss is given by:

$$D_{KL}\left(F_{Y_{o}},F_{Y_{s}}\right)=-log\left(z\right)+\frac{1}{2\sigma^{2}}\left[{\left(\mu_{S}-\mu_{2}\right)}^{2}-{\left(\mu_{S}-\mu_{1}\right)}^{2}\right],$$ (2)

where $\mu_{S}$ is the mean of the truncated normal distribution and equal to $\mu_{S}=E\left(y|y\geq y_{c}\right)=\mu_{1}+\sigma \left[\frac{\varphi \left(\left(y_{c}-\mu_{1}\right)/\sigma \right)}{1-\Phi \left(\left(y_{c}-\mu_{1}\right)/\sigma \right)}\right],$ and φ and Φ denote the *pdf (probability density function)* and *cdf (cumulative distribution function)* of a standard Gaussian random variable, respectively. By reordering some terms, Equation (2) can be alternatively formulated as:

$$D_{KL}\left(F_{Y_{o}},F_{Y_{s}}\right)=log\frac{1}{\text{Pr}\left(y>y_{c}\right)}+\frac{1}{2}\left\{\frac{{\left(S-R\right)}^{2}}{\sigma^{2}}-i^{2}\right\}$$ (3a)

$$=log\frac{1}{\text{Pr}\left(y>y_{c}\right)}+\frac{1}{2}\left\{i^{2}(h^{2}(h^{2}-2))\right\}$$ (3b)

where $S=\mu_{S}-\mu_{1}$ is the selection differential, $R=\mu_{2}-\mu_{1}$ is the selection response, and i=S/σ is the selection intensity. The second term on the right-hand side of Equation (3a) implies that when *R* approaches *S* (increased genetic gains) and the selection intensity increases, the divergence between the truncated distribution and the candidate’s distribution decreases. That is, $D_{KL}\left(F_{Y_{o}},F_{Y_{s}}\right)$ depends on the intensity of selection and is a decreasing function of $h^{2}$ (3b).

### Multivariate Kullback-Leibler (KL) loss function

In GS there are usually many traits of interest for which multivariate normality is assumed. As in the single-trait case, we can compute the divergence between two multivariate distributions and capture all the uncertainty in multivariate BVs. Then, assuming multivariate normality for phenotypic values of the traits under consideration, the KL divergence accounts for the association between traits, which improves and simplifies the decision procedure, given that all traits share some degree of association and are rarely independent.

Let $Y={\left(Y_{1},Y_{2},\ldots ,Y_{t}\right)}^{’}\in {\mathbb{R}}^{t}$ be the random vector of the phenotypic values of t traits of interest in the base population. We assume that $Y∼MVN\left(\mu_{1},P\right)$, where $\mu_{1}={\left(\mu_{1},\mu_{2},\ldots ,\mu_{t}\right)}^{’}$ is the vector of means in the parental population and P is a positive definitive variance-covariance matrix that captures the association between traits. Now, let $Y_{s}=\left(Y_{s1},Y_{s2},\ldots ,Y_{st}\right)’\in {\mathbb{R}}^{t}$ be the random vector resulting from left truncation at $y_{c}=\left(y_{c1},y_{c2},\ldots ,y_{ct}\right)’\in {\mathbb{R}}^{t}$. Then $Y_{s}∼TMVN\left(\mu_{1},P,y_{c}\right)$, where *TMVN* denotes the Truncated Multivariate Normal distribution. The *pdf* of $Y_{s}$ is

$$\pi \left(y|\mu_{1},P,y_{c}\right)={\left(2\pi \right)}^{-\frac{t}{2}}\;{\left|K\right|}^{-\frac{1}{2}}\exp\left\{-\frac{1}{2}\left(y-\mu_{1}\right)'P^{-1}\left(y-\mu_{1}\right)\right\}\left(\frac{1}{z}\right);\;$$

$y\in Y_{c}=\left\{y\in {\mathbb{R}}^{t}:y\geq y_{c}\right\},\;$ where the normalization factor is $z=Pr(y\geq y_{c})$. Now, let $Y_{o}={\left(Y_{o1},Y_{o2},\ldots ,Y_{ot}\right)}^{’}\in {\mathbb{R}}^{t}$ be the random vector of the phenotypic values of a candidate, and assume that $Y_{o}∼MVN\left(\mu_{2},P\right)$. Let’s define $S=\left(\mu_{s}-\mu_{1}\right){’}_{t\times 1}$ as the differential selection vector and $R=\left(\mu_{2}-\mu_{1}\right){’}_{t\times 1}=\mathrm{SG}P^{-1}$ as the vector of the selection response, where G is the genotypic covariance matrix. As in the univariate approach, the multivariate LF based on the multivariate KL divergence is given by (see Appendix A):

$$\;D_{KL}\left(F_{Y_{o}},F_{Y_{s}}\right)=\overset{\infty }{\underset{y_{c}}{\int }}\log\frac{TMVN(\mu_{1},P,y_{c})}{MVN(\mu_{2},P)}TMVN(\mu_{1},P,y_{c})dy$$ (4a)

$$=\log\left(z\right)+\frac{1}{2}S^{’}\left[{\left(I-GP^{-1}\right)}^{’}P^{-1}\left(I-GP^{-1}\right)-P^{-1}\right]S.\;\;$$ (4b)

Thus, as the phenotypic and genotypic covariance matrices tend to explain the same amount of variation and association between traits, $I-GP^{-1}=0$, the divergence between the parents’ distribution and the candidates’ distribution tends to decrease. Note that the expression $GP^{-1}\;$is the matrix equivalent of multi-trait heritability (the ratio of the genetic variance in the numerator and the phenotypic variance in the denominator); thus, when $GP^{-1}=I$, the heritability of each trait is 1 and R=S and **$\mu_{2}=\mu_{s}$**.

As in the univariate approach, the multivariate KL loss contains the term –log(z) that indicates that the joint probability of $Y_{o}$ falling above $y_{c}$ has less penalty.

**Figure B1.**
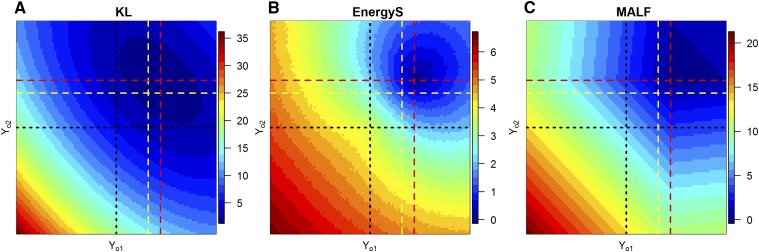
Bivariate representation of multivariate loss functions. A) Kullback-Leibler (KL), B) Energy Score (EnergyS), and C) Multivariate Asymmetric Loss Function (MALF). The base population is $N_{2}(\mu ,\;P)$, where μ=c(0,0)’ (black lines) and $P=\left[\begin{array}{cc} 2 & -1\\ -1 & 2 \end{array}\right]$. After truncation at $y_{0}=(1.3,\;1.3)’$ (yellow lines), the theoretical or desired mean of selected candidates is $\mu_{s}=(1.79,\;1.79)’$ (red lines). The candidates close to the region where each loss function is minimized will have minimum loss under the breeder’s preferences; those lines are the ones to advance in a selection program.

Note that P is expressed in terms of its inverse; therefore, those traits with high variance induce a lower penalty, and there is a compromise between the gain from selection and keeping the variability. KL divergence is an appropriate way of selecting because it not only captures the association between traits, but also assigns weights to each trait automatically, thus reducing subjectivism in GS. In addition, Equation (4b) implies that traits with higher variance will have priority in a GS program and will directly preserve the genetic variance as much as possible. Univariate and multivariate KL LFs are represented in Figure 1a (red lines) and Fig. B1a (Appendix B), respectively.

### The Continuous Ranked Probability Score and its generalization, the Energy Score

The Continuous Ranked Probability Score (CRPS) function is discussed in detail in Gneiting and Raftery (2007) and Hersbach (2000). The CRPS is a metric that reflects the distance between two random variables in terms of their cumulative distributions. The CRPS in GS acts by choosing candidate lines whose (cumulative) BV distributions are as close as possible to the hypothetical parental distribution that reflects the breeder’s preferences. Nevertheless, the main difference between KL divergence and CRPS is that the latter is less restrictive with lines whose distributions do not perfectly match the distribution that reflects our preferences, as can be seen in Figure 1a (green line).

Keeping the normality assumption, let $Y_{o}$ be a normal random variable with mean $\mu_{2}$ and variance $\sigma^{2}$; there is a closed form of this LF (Appendix A), which in our context is defined as

$$CRPS\left(F_{Y}{}_{o},\mu_{s}\right)=-\sigma \left[\frac{1}{\sqrt{\pi }}-2\varphi \left(\frac{\mu_{s}-\mu_{2}}{\sigma }\right)-\left(\frac{\mu_{s}-\mu_{2}}{\sigma }\right)\times \left(2\Phi \left(\frac{\mu_{s}-\mu_{2}}{\sigma }\right)-1\right)\right]$$ (5a)

$$=-\sigma \left[\frac{1}{\sqrt{\pi }}-2\varphi \left(\frac{\text{S}-\text{R}}{\sigma }\right)-\left(\frac{\text{S}-\text{R}}{\sigma }\right)\left(2\Phi \left(\frac{\text{S}-\text{R}}{\sigma }\right)-1\right)\right]$$ (5b)

$$=-\sigma \left[\frac{1}{\sqrt{\pi }}-2\varphi \left(i(1-h^{2})\right)-i(1-h^{2})\left(2\Phi (i(1-h^{2}))-1\right)\right]$$ (5c)

where φ and Φ denote the *pdf* and *cdf* of a standard Gaussian variable, respectively. Again, CRPS increases with the intensity of selection and is a decreasing function of heritability.

When the interest is in multiple traits, we can use the Energy Score (EnergyS), which generalizes the univariate CRPS (Gneiting and Raftery 2007; Székely and Rizzo 2013) and allows a direct comparison between multi-traits. The Energy Score in terms of a genomic selection goal is expressed as

$$ES\left(F_{Y}{}_{o},\mu_{s}\right)=E_{F}\|Y_{o}-\mu_{s}\|-\frac{1}{2}E_{F}\|Y_{o}-Y_{o}’\|$$ (6)

where ‖⋅‖ denote the Euclidean norm, $Y_{o}$ and $\mu_{s}$ were previously defined, and $Y_{o}’$ denotes an independent random vector with the same distribution as $Y_{o}$, *i.e.*, $F_{Y_{o}}$. In contrast to KL divergence, with the CRPS and the Energy Score we can avoid the normality assumption while holding the assumption that traits are on an interval scale. This is a big advantage given that frequently the traits of interest in GS are not Gaussian. The EnergyS is depicted in Fig. B1b (Appendix B).

### An asymmetric loss function in univariate and multivariate settings

At this point, the previous LFs that we adapted to the GS problem are all symmetric. Therefore, the penalties on the right- and left-hand side of the target at the same distance are equal. Nevertheless, it is natural to think that in conventional and GS plant breeding, those values of $Y_{o}$ greater than $y_{c}$ might be less penalized if the goal is to increase phenotypic values. Here is where an asymmetric LF plays a key role because it assigns very small penalties to those individuals whose BV distributions are to the right of the hypothetical parental distribution. One simple asymmetric LF is the linear-linear loss (LinLin), whose behavior is linear on both sides of the target; it has the α∈(0,1) term that induces different penalties (Berk 2011) and is the loss of the quantile regression. The LinLin LF is defined as

$$L\left(F_{Y}{}_{o},\alpha \right)=\left(\alpha -1\left(e<0\right)\right)e$$ (7)

where $e=\mu_{s}-\mu_{2}=S-R=\sigma i(1-h^{2})$; then this LF is also a decreasing function of $h^{2}$. LinLin loss is depicted in Figure 1a (blue line). Note that when α=0.5, the LinLin loss reduces to a symmetric linear LF.

In the multi-trait setting, we define $e=\left(\mu_{s}-\mu_{2}\right){’}_{t\times 1}=S-R=S(I-GK^{-1})$ as the vector of deviations of $\mu_{2}$ from $\mu_{s}$ and the multivariate LinLin function is 0 when phenotypic and genotypic matrices are identical, $GK^{-1}=I$. The *Multivariate Asymmetric Loss Function* (MALF) (Komunjer and Owyang 2011) is the generalization of LinLin LF. For GS purposes, it is expressed as

$$L_{2}\left(F_{Y}{}_{o},\mu_{s},\tau \right)=\left(∥e{∥}_{2}+\tau^{'}e\right)∥e{∥}_{2}$$ (8)

where $∥e{∥}_{2}={\left(e_{1}{}^{2}+e_{2}{}^{2}+\ldots +e_{t}{}^{2}\right)}^{\frac{1}{2}}$ is the Euclidean norm and τ controls the degree of asymmetry. In a simplified version with the $L^{1}-norm$, Equation (8) is expressed as

$$L_{1}\left(F_{Y}{}_{o},\mu_{s},\tau \right)=\left|e\right|+\tau^{'}e.\;$$ (9)

In the univariate case, letting τ=2α−1, Equation (9) reduces to twice the LinLin LF, *i.e.*, $L_{1}\left(e,\tau \right)=2L\left(e,\alpha \right)$. Both univariate and multivariate LinLin losses favor selecting those lines with distributions similar to the hypothetical parental distribution (which reflects the breeder’s preferences) as much as possible. The MALF is depicted in Fig. B1c (Appendix B).

### Posterior expected loss and their approximations

Each of the LFs proposed above can be used in conventional selection and/or GS to select the best lines according to our preferences. Below we briefly explain the formal approach used to evaluate posterior mean of the LFs proposed above. Given a prediction model (for example, Multi Trait Model (MTM) or Bayesian Ridge Regression (BRR) for a single trait), we have the joint posterior distribution

p(θ|y,X)∝Lik(θ|y, X)p(θ), (10)

where Lik(θ|y, X) is the likelihood function, and p(θ) is the prior distribution for θ, and elements of θ are an overall mean (μ), marker effects or any other effects (β), and the variance-covariance matrix (P) captures the association between traits (or $\sigma^{2}$, the phenotypic variance in a univariate framework), y is a matrix (or vector in a single-trait setting) containing phenotypic records, and X represents the incidence matrix according to the multi-trait or single-trait model.

Let $Y_{o}$ be the random vector of the phenotypic values of a candidate with a covariate vector of markers $x_{o}$ and distribution $F_{Y_{o}}(y_{o};\theta )$. The posterior predictive density corresponding to $F_{Y_{o}}$ is given by

$$f\left(y_{o}|x_{o},\;y,X\right)=\underset{\theta \in \Theta }{\int }f\left(y_{o}|\theta ,\;x_{o}^{'}\right)p\left(\theta |y,X\right)\partial \theta .$$ (11)

Hence, given the observed phenotypic and genomic data ($y,X,x_{o}$), whatever we can predict about the candidate is given by (11) and any choice of the candidates for selection must take into account the uncertainty described by $f\left(y_{o}|x_{o},\;y,X\right)$.

According to the Bayesian decision theory, the optimal choice is one that minimizes the posterior expected loss. In this case, once the phenotypical values, y, and the matrix of covariables, X, are observed, we have to average the LF over all the unknowns in the model, that is, θ and the observable but unknown phenotypic value of the *o*-th candidate. Given that $\mu_{s}$ is computed as a function of μ, **β** and P **(**or $\sigma^{2}$**)**, hereinafter we will denote all LFs described above as $L\left(F_{Y_{o}},\theta \right)$. Thus, the posterior expected value of the LF for candidate *o* is given by

$${\bar{L}}_{o}=\underset{y_{o}\in Y}{\int }\underset{\theta \in \Theta }{\int }L\left(F_{Y_{o}},\theta \right)f\left(y_{o}|\theta ,x_{o}^{'}\right)p\left(\theta |y,X\right)\partial \theta dy_{o}.$$ (12)

Then, for each *o* line, we have a posterior expected loss *${\bar{L}}_{o}$* and we will select those lines with the lowest posterior expected losses.

The expected value of the LFs given in (2), (5a) and (7), as well as their generalizations in the multivariate context (4b), (6), and (9) are approximated by Markov Chain Monte Carlo integration by taking m samples from the joint posterior distributions for $\mu_{1},\mu_{2},\mu_{s},\sigma^{2}$, $Y_{o}$ (in the univariate context) or for vectors $\mu_{1},\;\mu_{2},\mu_{s},P$, $Y_{o}$ (in the multivariate context).

### Application of univariate and multivariate loss functions in a wheat dataset

To illustrate the application of univariate and multivariate LFs in GS, we used 320 spring wheat lines with records on four traits: grain yield (GY), thousand-kernel weight (TKW), Zn and Fe concentrations in the grain (GZnC and GFeC, respectively) from CIMMYT’s biofortification breeding program and genotyped with DaRT markers (Velu *et al.* 2016). All traits are positively but low correlated; for example, GY had a correlation of 0.21 with TKW but zero with the other traits, whereas GZnC and GFeC had a 0.26 correlation.

For univariate LFs, we used Bayesian Ridge Regression (BRR) and the BGLR R package (de los Campos and Pérez Rodríguez 2015) in order to obtain posterior distributions of parameters and quantities of interest. We selected the top 10% (32 lines) of the wheat lines whose posterior expected losses were the minimum using the three univariate LFs discussed above (KL, CRPS, and LinLin). Results were contrasted with the top 10% based solely on punctual predicted BVs (Std), *i.e.*, by selecting individuals with the highest punctual predicted BVs. For multivariate LFs (KL, Energy Score and MALF), we fitted the MTM model (de los Campos and Grüneberg 2016). The aim here was to select the “best” lines whose performance across all traits is high. For asymmetric LFs (LinLin and MALF), we fixed the value of α (or τ) equal to 0.9 (according to selection pressure) in order to impose big penalties on those individuals far to the left of the target point; on the other hand, lower penalties were given to those that were equal or greater.

### Application of univariate and multivariate loss functions in a simulation scheme

Simulations were used to evaluate the three univariate and multivariate LFs discussed above. In the univariate scenario, we compared results obtained with the selection based on the standard method (Std). In the multivariate scenario, we contrasted results of different LFs. A recurrent selection scheme was used in which the best individuals were selected based on their merit regarding a single quantitative trait or three correlated quantitative traits, and measured through the LFs.

### Traits and heritabilities

In the univariate simulation, one trait was genetically simulated, where the gene effects were sampled from a gamma distribution with shape and scale parameters equal to two. Phenotypes were simulated by summing up all true genotypic values and adding a residual effect consistent with the expected heritability fixed at 0.5. That is, $y_{i}=\overset{p}{\underset{i=1}{\sum }}x_{ij}b_{j}+e_{i}=\text{TBV}s_{i}+e_{i}$, where p is the number of genes, and $x_{ij}$ is the genotype for the j-th gene of the i-line (coded as -1 and 1 for the two homozygotes, respectively). The environmental noise was sampled as $e_{i}∼N(0,\;\sigma_{g}^{2}(1-h^{2})/h^{2}\;)$, where $\sigma_{g}^{2}$ is the genetic variance.

In the multi-trait simulation, three correlated quantitative traits were genetically simulated assuming a full pleiotropic model. This was carried out by randomly sampling gene effects for all segregating sites from a multivariate normal distribution with zero mean and a previously stated variance-covariance, to ensure a genetic correlation at first generation of -0.37 between trait 1 (T1) and trait 2 (T2); a genetic correlation of 0.34 between traits 2 (T2) and 3 (T3); and a genetic correlation of -0.02 between T1 and T3. To mimic complex and simple quantitative traits, narrow-sense heritabilities of 0.3 and 0.6 were assumed for all traits. Hereinafter, we will always refer to the traits in terms of narrow-sense heritability ($h^{2}$), given that in the simulation scheme we simulated a purely additive model and did not include dominance effects.

### Selection cycles

The forward-in-time component represented ten cycles of a classic recurrent selection scheme in which the breeder has the ability to select based on the genomic predictions of the breeding values.

In the multivariate LFs framework, genomic predictions of the evaluated lines were carried out using the Multi-Trait Model (MTM) (de los Campos and Grüneberg 2016), where 70% and 30% of the lines were used as the training and testing sets (candidates), respectively. For single-trait simulation, genomic predictions were made using Bayesian Ridge Regression (BRR).

For multi-trait simulation, the simulated phenotypic values were modeled assuming an intercept for each trait as a fixed effect and the predicted breeding value for each line in each trait as random. As in the wheat dataset, we fitted the same MTM model for the multi-trait simulation. In both the univariate and multi-trait simulation, posterior distributions of the predicted breeding values of the candidate lines were ranked according to each selection process (the univariate and multivariate LFs described above). For all LFs described above, approximations to the posterior expected loss were obtained considering 10,000 MCMC samples after a burn-in period of 30,000 samples. For univariate simulation, the top 10% and the top 30% of the lines in the candidate set were selected via their minimum posterior expected losses, while in the multi-trait simulation scheme, the top 10% of the lines in the candidate set were selected with minimum posterior expected losses.

In the LinLin LF and its generalization MALF, we fixed the value of α or τ equal to 0.9 or 0.7 (depending on whether the selection pressure was 10% or 30%) in order to impose big penalties on those individuals in the candidate set that were on the far left-hand side of the target point, while lower penalties were given to those that were equal or greater.

Results are presented as summaries of 20 replicates for each heritability, selection pressure and LF scenario. Individuals selected as parents will have the highest possible BV and the lowest possible value of the corresponding LFs. The simulations were implemented in a C++ program that was compiled, linked and executed within the R version 3.3.3 (R Development Core Team 2017) through the facilities provided by the Rcpp package (Eddelbuettel and Francois 2011).

### Data Availability

The durum wheat data including clean imputed markers and phenotypic information for the traits can be found in the following link: http://genomics.cimmyt.org/Decision_theory_GS/

## Results

### Univariate loss functions for wheat data

**Figure 2:**
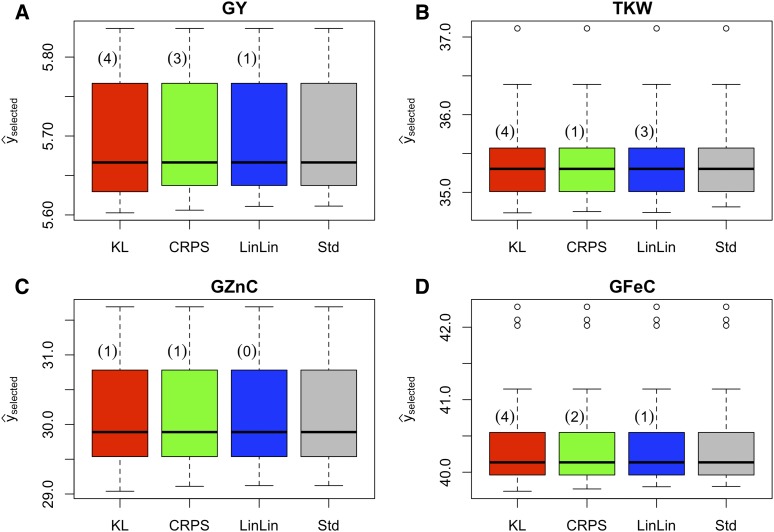
Univariate real data. Boxplots of estimated breeding values for a real wheat dataset (with four traits) of the top 10% of selected lines with three univariate loss functions Kullback-Leibler (KL), Continuous Ranked Probability Score (CRPS) and Linear-Linear loss (LinLin), and breeding values of the lines selected under the standard method (Std) for A) Grain Yield (GY), B) thousand-kernel weight (TKW), C) Zn concentration in grain (GZnC), and D) Fe concentration in grain (GFeC). Values in parentheses are the lines that the loss functions selected but the Std did not.

The boxplots in Figure 2a-d correspond to the predictive BVs of the top 10% spring wheat lines using univariate LFs (KL, CRPS, and LinLin) and the Std method (not using LFs) for the four traits. In general, no changes in the mean of selected candidates of the traits are found in the top 10% of the wheat lines because only a few of them (numbers in parentheses) changed for each LF, as compared with the Std method. Note that we only evaluated the selection differential because we could not cross the selected lines to compute the selection response as we did in the simulation study. In fact, KL was the only LF that had four different selected lines for traits GY, TKW and GFeC and only one line for trait GZnC. In contrast, LinLin loss selected almost the same lines as the Std method, given that traits GY and GFeC only diverged in one line, trait TKW diverged in three individuals, and in trait GZnC no different individuals were selected. CRPS LF performed between KL and LinLin by selecting 3, 1, 1, and 2 lines for traits GY, TKW, GZnC and GFeC, respectively, which were different than the lines selected by Std. Despite the fact that only a few lines with BV ranked differently under the LFs *vs.* the Std method, the overall effect of those different selected lines can change over subsequent selection cycles.

### Multivariate loss functions for wheat data

**Figure 3:**
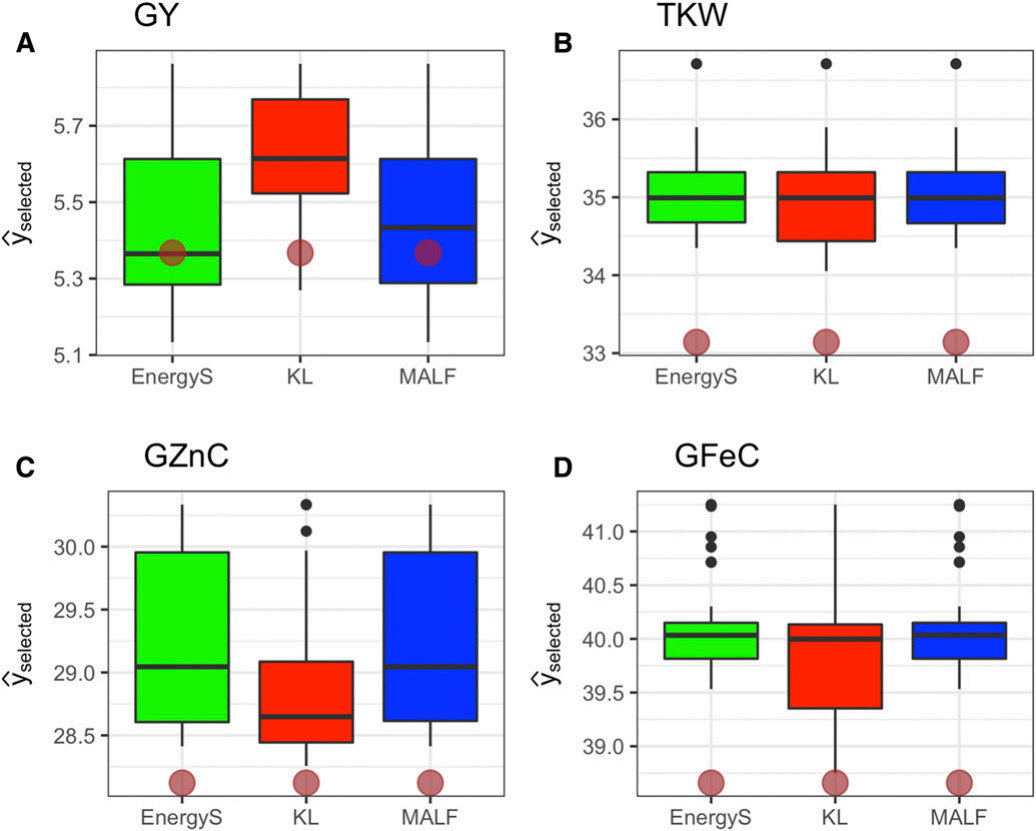
Multivariate real data. Boxplots of estimated breeding values for a real spring wheat dataset of the top 10% of selected lines for four traits according to the multivariate loss functions Kullback-Leibler (KL), Energy Score (EnergyS) and Multivariate Asymmetric Loss Function (MALF) for A) grain yield (GY), B) thousand-kernel weight (TKW), C) Zn concentration in grain (GZnC), and D) Fe concentration in grain (GFeC). Brown dots indicate the mean of all lines.

Figure 3a-d depicts the boxplot of predictive BVs of selected individuals using multivariate LFs, EnergyS, KL and MALF. The selected candidates are the top 10% of lines selected for the four traits.

For the complex trait GY, which has a 0.21 sample phenotypic correlation with trait TKW and zero correlation with the other traits, the KL was the best LF and the mean of the selected lines was superior to the population mean. The mean GY of the top 10% of the lines developed using MALF ranked second and the top 10% of the lines selected by EnergyS multivariate LF were the worst of the three LFs that were not better than the mean of the base population. For trait TKW, the top 10% of the best lines in the three LFs were similar and superior to the mean of the original population. For trait GZnC, EnergyS and MALF LFs produced the best lines, which were slightly better than those produced by the KL LF. Finally, for trait GFeC, the three LFs produced the top 10% of lines with similar Fe concentration in the grain.

The best LF was the KL loss, given that it selected individuals that, on average, performed better than the estimated population mean in all traits. Results of Hotelling’s two sample T2-tests indicate that the mean of the selected individuals between KL and EnergyS was statistically different at a 0.05 confidence level for error type I. The same test suggested no differences when contrasting KL *vs.* MALF and MALF *vs.* Energy. On the other hand, we computed the percentage of lines that do not intersect in each combination of LFs. For example, between KL and EnergyS LFs, 38% (12 of 32) of the lines are different between both LFs. Between KL and MALF, 34% (11 of 32) of the lines are different. In contrast, EnergyS *vs.* MALF had only 3% (1 of 32) of different selected lines between both LFs. By crossing the selected candidates from each of the LFs and repeating the procedure over selection cycles, we would expect a gain in the mean of all traits, with better performance, on average, from the lines selected under the KL LF.

### Univariate loss function for simulation data

In the following results of the univariate and multivariate simulation framework, the population mean for each cycle was standardized according to $(\mu_{i}-\mu_{1})/\sigma_{1}=R_{i}/\sigma_{1}$. This is a no-dimensional quantity where $\mu_{i}$ represents the population mean in cycle i, $\mu_{1}$ is the population mean of the first cycle, $R_{i}$ is the selection response in cycle i with respect to the first, and $\sigma_{1}$ is the population standard deviation of cycle 1. For the variance population, we scaled the variances of cycle i by the variances of the first cycle, *i.e.*, $\sigma_{i}^{2}/\sigma_{1}^{2}$.

**Figure 4:**
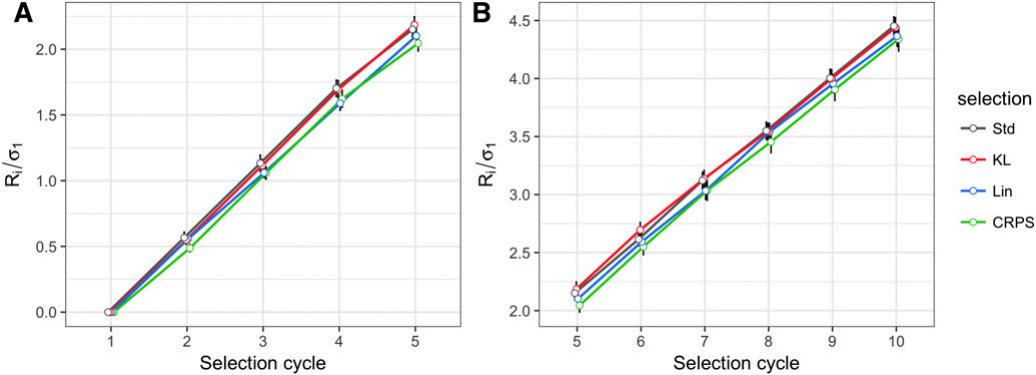
Results of the univariate simulation study. Standardized selection response $R_{i}/\sigma_{1}=(\mu_{i}-\mu_{1})/\sigma_{1}\;$for breeding cycles 1 to 5 are illustrated in A), while cycles 5 to 10 are in B). In each selection cycle, the top 10% of lines with minimum posterior expected losses were selected using the Kullback-Leibler loss function (KL), the Continuous Ranked Probability Score (CRPS), the Linear-Linear loss function (LinLin), and the standard method (Std). Selected lines were crossed in each cycle to recover the population size for upcoming selection cycles. $\mu_{i}$ and $R_{i}$ represent the population mean and the selection response, respectively, in cycle i; $\mu_{1}$ and $\sigma_{1}$ are the population mean and the population standard deviation, respectively, in cycle 1. The black vertical lines indicate the standard error of $R_{i}/\sigma_{1}$ under 20 replications of the simulation study.

**Figure 5:**
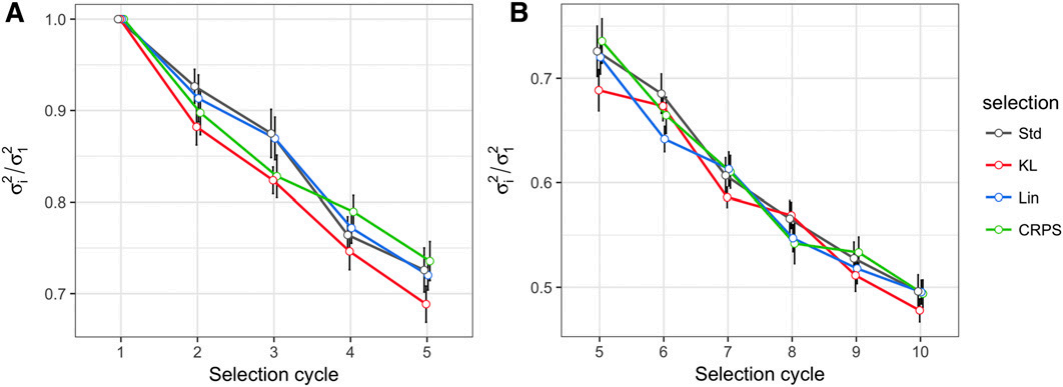
Results of the univariate simulation study. Scaled population variance $\left(\sigma_{i}^{2}/\sigma_{1}^{2}\right)$ for breeding cycles 1 to 5 are illustrated in A), while cycles 5 to 10 are in B). In each selection cycle, the top 10% were selected using the Kullback-Leibler (KL), the Continuous Ranked Probability Score (CRPS), and the Linear-Linear (LinLin) loss functions, and lines selected under the standard method (Std). Selected lines were crossed at each cycle to recover the population size for upcoming selection cycles. $\sigma_{i}^{2}$ and $\sigma_{1}^{2}$ are the population variance in cycle i and cycle 1, respectively. The black vertical lines indicate the standard error of $\sigma_{i}^{2}/\sigma_{1}^{2}$ under 20 replications of the simulation study.

**Figure B2.**
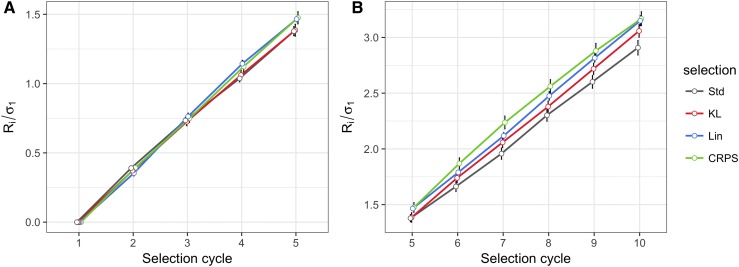
Results of the univariate simulation study. Standardized selection responses $R_{i}/\sigma_{1}=(\mu_{i}-\mu_{1})/\sigma_{1}\;$for breeding cycles 1 to 5 are illustrated in A), while in B) are cycles 5 to 10. In each selection cycle, the top 30% with minimum posterior expected losses were selected using the Kullback-Leibler loss function (KL), the Continuous Ranked Probability Score (CRPS), the Linear-Linear loss function (LinLin), and the standard method (Std). Selected lines were crossed at each cycle to recover the population size for upcoming selection cycles. $\mu_{i}$ and $R_{i}$ correspond to the population mean and the selection response, respectively, in cycle i; $\mu_{1}$ and $\sigma_{1}$ are the population mean and the population standard deviation, respectively, in cycle 1. The black vertical lines indicate the standard error of $R_{i}/\sigma_{1}$ under 20 replications of the simulation study.

**Figure B3.**
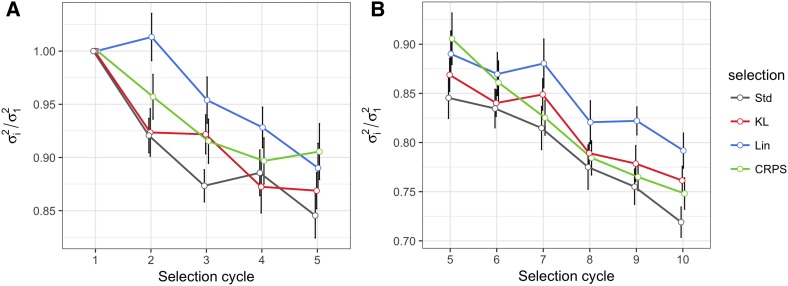
Results of the univariate simulation study. Scaled population variance $\left(\sigma_{i}^{2}/\sigma_{1}^{2}\right)$ for breeding cycles 1 to 5 are illustrated in A), while in B) are cycles 5 to 10. In each selection cycle, the top 30% were selected using the Kullback-Leibler loss function (KL), the Continuous Ranked Probability Score (CRPS), the Linear-Linear loss function (LinLin), and lines selected under the standard method (Std). Selected lines were crossed at each cycle to recover the population size for the upcoming selection cycles. $\sigma_{i}^{2}$ and $\sigma_{1}^{2}$ are the population variance in cycle i and cycle 1, respectively. The black vertical lines indicate the standard error of $\sigma_{i}^{2}/\sigma_{1}^{2}$ under 20 replications of the simulation study.

Figures 4a-b show the trends of the average standardized population mean for the univariate simulation scheme separating cycles 1-5 and cycles 5-10 for better visualization. The scale population variance is depicted in Figures 5a-b. These figures summarize the results when selecting the top 10% of candidates with minimum posterior expected losses. There were no significant changes in the population means and population variances when comparing LFs *vs.* the conventional (Std) selection, *i.e.*, LFs performed similarly to Std. In contrast, when the selected proportion was 30% of the candidates, there was a slight improvement using LFs as the selection program advanced, as depicted in Figs. B2a-b (Appendix B) for the standardized population mean and in Figs. B3a-b (Appendix B) for the scaled population variance.

**Figure 6:**
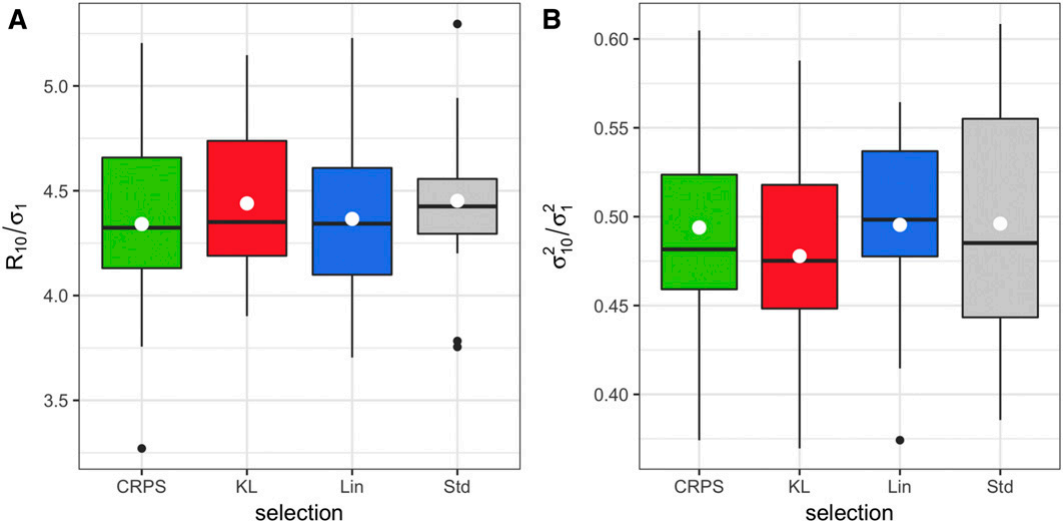
Results of the univariate simulation study at the 10^th^ selection cycle for 10% of the selected lines. A) boxplots of the standardized selection response; B) boxplots of the scale population variance using the Kullback-Leibler (KL), the Continuous Ranked Probability Score (CRPS), the Linear-Linear (LinLin) loss functions, and lines selected under the standard method (Std). The boxplots illustrate the mean (white dots) and median (black middle line) of 20 replications of the simulation study. $R,\sigma^{2},\sigma_{1}^{2}$ and $\sigma_{1}$ were defined in Figs. 4 and 5. Sub-indices refer to the 10^th^ selection cycle.

**Figure B4.**
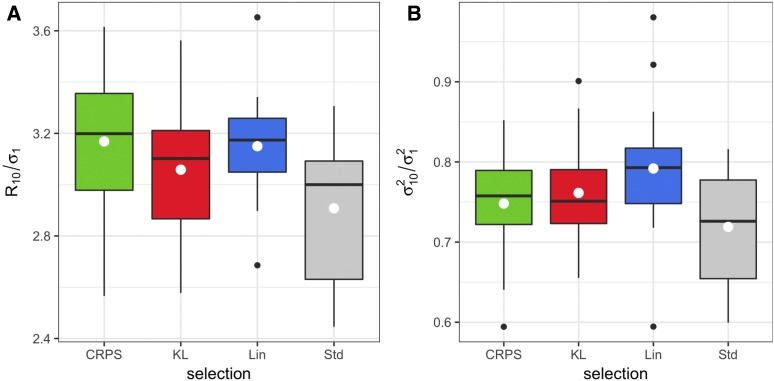
Results of the univariate simulation study at the 10^th^ selection cycle for 30% of selected lines. A) Boxplots of the standardized selection response; B) boxplots of the scale population variance. The boxplots illustrate the mean (white dots) and median (black middle line) of 20 replications of the simulation study. $R,\sigma^{2},\sigma_{1}^{2}$ and $\sigma_{1}$ were defined in Figs. B2 and B3. Sub-indices refer to the 10^th^ selection cycle.

The boxplots in Figure 6a and Figure B4a (Appendix B) depict the mean of the 10-th selection cycle when the proportions of selected individuals were 10% and 30%, respectively. When 10% were selected, no differences were found in terms of the standardized response to selection between LFs and the standard method. In contrast, when 30% were selected, the LFs performed better than the standard method in terms of the average response to selection in the 10-th cycle. Table 1a gives the *t*-test for comparing differences in the mean when selecting 10% of the lines under the univariate LFs *vs.* the standard method (Std), whereas Table 1b shows the same results but for the 30%. There were significant differences among the population means of lines selected under LF *vs.* those selected without employing any LF for the 30%.

**Table 1:** Simulation univariate study. Student t-test of mean and variance differences between the lines selected by the univariate Kullback-Leibler (KL), Continuous Ranked Probability Score (CRPS) and Linear-Linear (LinLin) loss functions *vs.* lines selected under the standard selection method (Std), after 20 replications of the simulated breeding program. The selected proportions were the top 10% and top 30% of the candidates, and the means and variances were compared at the 10^th^ selection cycle

contrast	a) mean of top 10%			b) mean of top 30%		
	*t*	df	p-value	*t*	df	p-value
CRPS *vs.* Std	−0.85	36	0.4	2.9	38	0.006*
KL *vs.* Std	−0.11	38	0.914	1.7	37	0.088
Lin *vs.* Std	−0.73	38	0.469	3.1	34	0.004*

contrast	c) variance of top 10%			d) variance of top 30%		
	*t*	df	p-value	*t*	df	p-value
CRPS *vs.* Std	−0.1	36.6	0.917	1.3	38	0.198
KL *vs.* Std	−0.9	33.8	0.355	2	37.8	0.052*
Lin *vs.* Std	0	34.5	0.973	3.1	37.2	0.003*

The results of the variance of the final populations in cycle 10^th^ are shown in Figure 6b and Figure B4b (Appendix B) for 10% and 30% of the selected individuals, respectively. When 10% were selected, the boxplot in Figure 6b does not show any difference in the variance obtained under the LFs *vs.* the variance of the population using the standard method. However, for the 30%, there were substantial differences between variances of populations selected under the LFs compared with those selected based solely on the mean of the breeding values (Figure B4b, Appendix B). Tables 1c and 1d show the *t*-test for comparing the mean of the population variance at the 10-th selection cycle when selecting 10% and 30% of the individuals. The only case where the mean of the population variance of the lines selected under the LF was statistically higher than the mean of the population variance of the lines obtained using the standard method was for KL and LinLin LFs when 30% of the individuals were selected.

### Multivariate loss functions for simulated data

#### The population mean:

**Figure 7:**
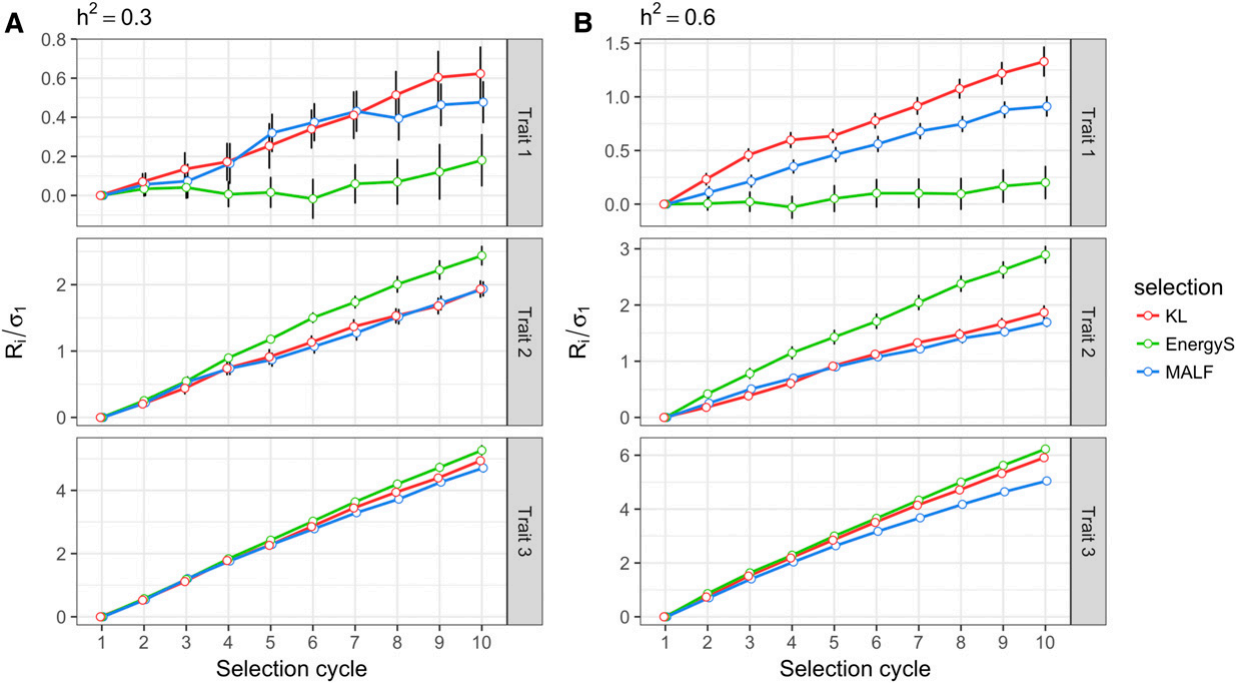
Results of the multivariate simulation study. A) Standardized population mean $(\mu_{i}-\mu_{1})/\sigma_{1}=R_{i}/\sigma_{1}$ for the breeding cycles when heritability for all traits was fixed at 0.3, and B) standardized population mean when heritability was 0.6 for all traits. In each selection cycle, the top 10% of candidates were selected using the multivariate loss functions: Kullback-Leibler (KL), Energy Score (EnergyS) and Multivariate Asymmetric Loss Function (MALF) to recover the population size for the upcoming breeding cycles. $\mu_{i}$ and $R_{i}$ correspond to the population mean and the selection response, respectively, in cycle i; $\mu_{1}$ and $\sigma_{1}$ are the population mean and the population standard deviation, respectively, in cycle 1. The black vertical lines indicate the standard error of $R_{i}/\sigma_{1}$ under 20 replications of the simulation study.

Figure 7 depicts the changes in the standardized population mean for each trait when the heritabilities for all traits were fixed at 0.3 (Figure 7a) and 0.6 (Figure 7b). For T1 with a negative correlation with T2 and negligible association with T3, the KL and MAFL LFs performed similarly with respect to the population mean throughout the breeding cycles, and their performance was better than the performance of the lines selected under EnergyS. The mean percentage differences computed for the 10^th^ cycle with respect the first cycle for both heritability cases are shown in Table 2a. For trait T1, KL gained 1.583% *vs.* 1.211% of the MALF LF for a heritability of 0.3. On the other hand, EnergyS had a conservative gain of 0.462%. For the second trait (T2), the three LFs had positive performances. EnergyS had the best performance, with a final gain of 8.224% in the 10^th^ cycle for a heritability of 0.3. In second and third place were MALF and KL with gains of 6.493% and 6.441%, respectively. Although the KL and MALF LFs had the smallest gains, the sign of the gains was positive for all selection cycles. It is important to note that the correlation between T1 and T2 was -0.37 and negligible between T1 and T3. Finally, for trait T3 with a heritability of 0.3, the performance of the population mean throughout the breeding cycles was similar for all three LFs. The percentages of gain with respect to the first cycle confirm these results. In descending order, the gains were EnergyS (6.14%), KL (5.762%) and MALF (5.499%) for a heritability of 0.3 (Table 2a).

**Table 2:** Simulation multivariate study. Means of percentage differences of population means a) and for population variance b) in the 10^th^ breeding cycle with respect to the first cycle for trait 1 (T1), trait 2 (T2) and trait 3 (T3) for lines selected under three multivariate loss functions Kullback-Leibler (KL), Energy Score (EnergyS) and Multivariate Asymmetric Loss Function (MALF) (standard errors are in parentheses). Heritability of 0.3 and 0.6 for all traits

	a)				b)		
	Loss	T1	T2	T3	T1	T2	T3
$h^{2}=0.3$	KL	1.583 (0.343)	6.441 (0.387)	5.762 (0.120)	-47.886 (1.678)	48.128 (1.506)	–51.181 (1.370)
	EnergyS	0.462 (0.332)	8.224 (0.477)	6.140 (0.173)	-47.533 (1.146)	-47.584 (1.372)	-50.287 (1.327)
	MALF	1.211 (0.264)	6.493 (0.344)	5.499 (0.139)	-47.437 (1.221)	-47.928 (1.496)	-47.820 (1.352)
$h^{2}=0.6$	KL	3.318 (0.327)	6.206 (0.403)	6.935 (0.118)	-44.532 (1.672)	-43.975 (1.341)	-49.588 (1.509)
	EnergyS	0.506 (0.375)	9.819 (0.500)	7.291 (0.139)	-45.256 (1.550)	-46.618 (1.456)	-48.025 (1.345)
	MALF	2.347 (0.232)	5.721 (0.239)	5.842 (0.121)	-44.977 (0.811)	-45.451 (0.827)	-44.910 (1.271)

When we fixed the heritabilities for all traits at 0.6, the results were similar to those described above. Table 2a shows the mean of the percent differences computed for the population mean in the 10^th^ cycle, taking the mean in the first cycle as a reference. For T1, those differences were, in descending order, 3.318% (KL), 2.347% (MALF) and 0.506% (EnergyS). For T2, EnergyS (9.819%) achieved the best performance for the population mean, followed by the KL LF (6.206%) and finally, MALF (5.721%). All LFs performed similarly for T3, although the EnergyS and KL LFs had the highest gains: 7.291% and 6.935%, respectively. They were followed very closely by MALF, with 5.842% of the cumulative gains at the end of the simulated selection program.

#### The population variance:

**Figure 8:**
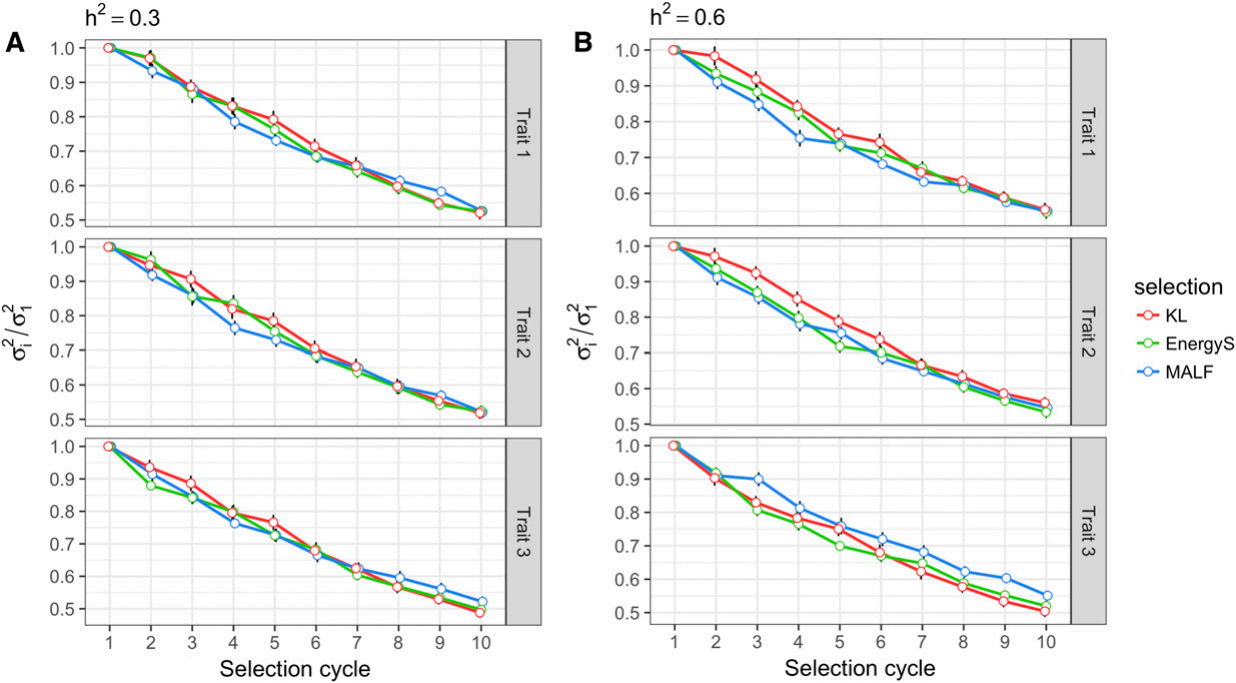
Results of the multivariate simulation study. A) Scale population variance $\left(\sigma_{i}^{2}/\sigma_{1}^{2}\right)$ for the breeding cycles when heritability for all traits was fixed at 0.3, and B) scale population variance when heritability was 0.6 for all traits. In each selection cycle, the top 10% of candidates were selected using the multivariate loss functions: Kullback-Leibler (KL), Energy Score (EnergyS) and Multivariate Asymmetric Loss Function (MALF) to recover the population size for the upcoming breeding cycles. $\sigma_{i}^{2}$ and $\sigma_{1}^{2}$ are the population variances for cycle i and cycle 1, respectively. The black vertical lines indicate the standard error of $\sigma_{i}^{2}/\sigma_{1}^{2}$ under 20 replications of the simulation study.

Figure 8 shows the averages of the scaled population variance for all traits in each selection cycle when the heritabilities for all traits were set at 0.3 (Figure 8a) and 0.6 (Figure 8b). Note that the decrease in the population variance was similar in all cases (trait-LFs combinations). Table 2b shows the mean differences in the percentages of population variance (not scaled) for each trait, computed for the 10^th^ breeding cycle taking the value of the first cycle as a reference. For T1 and a heritability of 0.3, these differences were, in descending order, -47.437% (MALF), -47.533% (EnergyS), and -47.886% (KL) (Table 2b). For T2 and 0.3 heritability, the decreases were -47.584% (EnergyS), -47.928% (MALF), and -48.128% (KL). Finally, the decreases in genetic variance for T3 and a 0.3 heritability for the LFs were -47.820% (MALF), -50.287% (EnergyS), and -51.181% (KL) (Table 2b). In general, these results show that the selection of parents does not kill more variance between LFs, although LFs reported different positive gains for the population mean of all traits.

When we fixed the heritability at 0.6 for all traits, the mean of population variances performed similarly for all traits, reinforcing the idea that the LFs proposed in this research do not kill more variance between them, as shown in Figure 8b. The losses in variance percentages (not scaled) computed in the last cycle with respect to the first selection cycle show that, for T1, the reductions in genetic variance were -44.532% (KL), -44.977% (MALF), and -45,256% (EnergyS) (Table 2b). For T2, the reductions in genetic variance were -43.975% (KL), -45.451% (MALF), and -46.618% (EnergyS). Finally, for T3, the reductions in genetic variance were -44.910% (MALF), -49.588% (KL), and -48.025% (EnergyS). In summary, all multivariate LFs described in this study performed similarly in reducing the genetic variance as the selection program advanced.

## Discussion

The objective in this study was to propose a formal methodology to select the best performing parents for future selection cycles in a genomic selection program. In a decision theory problem, LFs are the mechanism to penalize our potential decisions. In GS, LFs reflect our preferences for individuals with the desired characteristics to ensure the highest genetic progress. This approach is advantageous mainly when selection is conducted in multi-trait settings, where some of the traits are either positively or negatively correlated. Therefore, losses may be interpreted as deviations from our goal of improving genetic progress. Lines with posterior predictive distributions that are closer to the theoretical distribution will have minimum losses.

For this reason, we proposed three univariate LFs (Kullback-Leibler, CRPS and LinLin) and their multivariate generalizations (Kullback-Leibler, Energy Score, and MALF) to assist plant breeders when selecting the best performing parents for future selection cycles from a candidate set used in GS. The univariate and multivariate, symmetric and asymmetric LFs were presented as decreasing functions of the heritability of the traits(s). From this perspective, deviations between the distributions of candidate parents and the theoretical distribution that reflects the plant breeder’s preferences are seen as divergences. The previous sections presented the results obtained using a real dataset (wheat lines) during one selection cycle, and in a simulation study scheme under the univariate and multivariate LFs (single-trait and multi-trait). When selecting a single trait, we compared the performance of the LFs with the performance of the standard method of selection. The standard selection method ranks individuals based on punctual predictions of BVs. In the multi-trait simulation, we compared the performance of multivariate LFs.

The results of the univariate simulation were encouraging given that LFs performed better than the conventional method when the selection pressure was not too restrictive (30%). In the less favorable scenario, the LFs performed as well as the GS method that does not employ LFs (Std). These univariate results allow us to generalize the use of LFs to the multi-trait framework. In the multi-trait problem, the LFs can be considered as methods that balance the gains simultaneously. However, this does not mean that LFs can attain the maximum possible gains for each trait, such as when selection operates on only one trait at a time.

Addressing selection as a decision theory problem faces the restriction of multi-trait *vs.* single-trait selection; when some traits are negatively correlated, the selected trait might reach its optimum value while the other traits stay the same or move away from their optimum values. It is important to note that our methodology allows researchers to control parental distribution and thus induce selection that favors some traits over others and restricts the selection of individuals for particular traits, as selection indices do when using restrictive selection indices. For instance, suppose that for T1, we do not desire gains between selection cycles; it is enough to leave without truncation the distribution for such a trait ($y_{c}=\pm \infty$).

As previously stated, in the simulation study we induced T1 and T2 with a negative correlation; this means that as the phenotypic values of T1 increase, the phenotypic values of T2 decrease whenever the selection works in favor of T1, and vice versa. In the case of T2 and T3, the induced correlation was positive, whereas T1 and T3 were independent of each other (no correlation). This setting was purposely chosen to show that our methodology works even in the worst scenario. Results indicate that LFs worked in favor of all traits, in spite of their negative correlations; the EnergyS worked better for T2 and T3 than for T1, and in that sense, it is the least promising of the three multivariate LFs. Further research on comparing the performance of LFs *vs.* selection indices (SI) will be useful. Using SI to calibrate economic weights and obtain simultaneous gains in (negatively) correlated traits is cumbersome and economic analyses should be done. However, in multivariate LF, this is done automatically.

On the other hand, the population means for T2 obtained with KL and MALF showed a lower performance than those obtained with EnergyS, which is understandable given the good performance in T1 under both LFs. The KL and MALF chose those candidates whose BVs ensured gains in all traits. Therefore, for T2, EnergyS had a small but significant gain *vs.* MALF and KL loss, which is no surprise since it was at the expense of sacrificing the gains in T1. For T3, the LFs had similar performances, with small but significant differences between them. This is illustrated in Figure 7a-b. It should be pointed out that although the LF reported increases in the population means of the three traits (for the two heritability values), the population genetic variance decreased at the same rate for all LFs, as depicted in Figure 8a-b.

In summary, we propose a formal approach for selecting the best performing parents for upcoming selection cycles. Our approach is based on Bayesian decision theory to construct divergences (LFs) that score candidates. Expected posterior losses take into account uncertainties about parameters and predictions involved in regression models. Our results show that using the proposed LFs when selecting a single trait can improve the response to selection while preserving the population variance. In the multi-trait framework, the advantages of LFs are evident. The population means of all traits under consideration showed positive gains, even though two of them were negatively correlated. We believe that selection based on LFs is more convenient than selection based solely on BVs, given that in LFs the weights for each trait are calculated automatically. It is easier to fix a truncation point than to calibrate the economic weights used in selection indices. These results were analogous for both complex and simple traits.

Finally, the Bayesian decision theory using several loss functions studied in this research and applied to GS-assisted breeding can be extended and used in conventional plant and animal breeding methods. Loss functions described in this study can be employed in data from progeny trials in plant and animal breeding to rank the selected individuals based not only on the adjusted means, but also on the loss function for a single trait or multiple traits. As shown in the long-term simulation study, changes in the rank of a few individuals can change the final response to selection after several selection cycles.

## Conclusions

We proposed applying a formal methodology within the decision theory framework, to the problem of selecting in the single- and multi-trait context, when applying genomic selection in plant breeding. Therefore, we discussed a theoretical justification and then proposed three univariate LFs (Kullback-Leibler, CRPS, and LinLin) and their corresponding generalizations in a multivariate setting (Multivariate Kullback-Leibler, Energy Score and MALF) with a theoretical derivation that expresses the LFs in terms of the heritability of the trait(s). We performed an example on a real dataset during one selection cycle for univariate and multivariate LFs, and in a simulation study of a genomic selection program in order to compute the population mean and variance throughout the breeding cycles. We contrasted our results with those obtained using the standard selection method in a single-trait scenario (selection based on predictions of punctual breeding values). Our results suggest that it is possible to obtain better performance in a long-term breeding program in the single-trait context by selecting 30% of the best individuals in each cycle. For the multi-trait approach, our results show that the population mean for all traits under consideration had positive gains, even though two of the traits were negatively correlated. Although each multivariate LF performed very well in the population means, the corresponding population variances were not statistically different.

## Appendix A

### Univariate Kullback-Leibler loss derivation

$$\begin{array}{l} KL\left(F_{Y_{o}},F_{Y_{s}}\;\right)=\int_{y_{c}}^{\infty }log\frac{N_{T}(\mu_{1},\sigma^{2},y_{c})}{N(\mu_{2},\sigma^{2})}N_{T}(\mu_{1},\sigma^{2},y_{c})dy\\ =\int_{y_{c}}^{\infty }\log N_{T}(\mu_{1},\sigma^{2},y_{c})N_{T}(\mu_{1},\sigma^{2},y_{c})dy-\int_{y_{c}}^{\infty }\log N(\mu_{1},\sigma^{2})N_{T}(\mu_{1},\sigma^{2},y_{c})dy\\ =-\log\left(z\right)-\frac{1}{2\sigma^{2}}\int_{y_{c}}^{\infty }{\left(y-\mu_{1}\right)}^{2}N_{T}(\mu_{1},\sigma^{2},\;y_{c})dy+\frac{1}{2\sigma^{2}}\int_{y_{c}}^{\infty }{\left(y-\mu_{2}\right)}^{2}N_{T}(\mu_{1},\sigma^{2},\;y_{c})dy\\ =\cdot \cdot \cdot \\ =-\log\left(z\right)-\frac{1}{2\sigma^{2}}\left(V_{S}+\mu_{S}^{2}-2\mu_{1}\mu_{S}+\mu_{1}^{2}\right)+\frac{1}{2\sigma^{2}}\left(V_{S}+\mu_{S}^{2}-2\mu_{2}\mu_{S}+\mu_{2}^{2}\right)\\ =-\;\text{log}\left(z\right)+\frac{1}{2\sigma^{2}}\left\{{\left(\mu_{S}-\mu_{2}\right)}^{2}-{\left(\mu_{S}-\mu_{1}\right)}^{2}\right\} \end{array}$$

where $z=1-\text{Φ}(\frac{y_{c}-\mu_{1}}{\sigma })$, and $\mu_{S}$ and $\;V_{S}$ denote the mean and the variance, respectively, when truncation at $y_{c}$ occurs.

### Multivariate Kullback-Leibler loss derivation

$$\begin{array}{l} KL\left(F_{Y_{o}},F_{Y_{s}}\right)\\ =\int_{y_{c}}^{\infty }\log MVN_{T}(\mu_{1},P,y_{c})\;\;MVN_{T}(\mu_{1},P,y_{c})dy-\int_{y_{c}}^{\infty }\log MVN(\mu_{2},P)\;MVN_{T}(\mu_{1},P,y_{c})dy\\ =\int_{y_{c}}^{\infty }\left[-\log\left(z\right)-\frac{t}{2}\log\left(2\pi \right)-\frac{1}{2}\log\left(\det\left(P\right)\right)-\frac{1}{2}{\left(y-\mu_{1}\right)}^{\prime }P\left(y-\mu_{1}\right)\right]MVN_{T}(\mu_{1},P,y_{c})dy\\ -\int_{y_{c}}^{\infty }\left[-\frac{t}{2}\log\left(2\pi \right)-\frac{1}{2}\log\left(\det\left(P\right)\right)-\frac{1}{2}{\left(y-\mu_{2}\right)}^{\prime }P\left(y-\mu_{2}\right)\right]MVN_{T}(\mu_{1},P,y_{c})dy\\ =-\log\left(z\right)-\frac{1}{2}\int_{y_{c}}^{\infty }{\left(y-\mu_{1}\right)}^{\prime }P\left(y-\;\mu_{1}\right)MVN_{T}(\mu_{1},P,y_{c})dy\\ +\frac{1}{2}\int_{y_{c}}^{\infty }{\left(y-\mu_{2}\right)}^{\prime }P\left(y-\;\mu_{2}\right)MVN_{T}(\mu_{1},P,y_{c})dy\\ =\cdot \cdot \cdot =-\log\left(z\right)+\frac{1}{2}\left\{{\left(\mu_{s}-\mu_{2}\right)}^{\prime }P^{-1}\left(\mu_{s}-\mu_{2}\right)-{\left(\mu_{s}-\mu_{1}\right)}^{\prime }P^{-1}\left(\mu_{S}-\mu_{1}\right)\right\} \end{array}$$

with $z={\left(2\;\pi \right)}^{t/2}{\left(\det\left(P\right)\right)}^{-1/2}\int_{y_{c}}^{\infty }\text{exp}\left\{\frac{1}{2}{\left(y-\mu_{1}\right)}^{\prime }P^{-1}\left(y-\mu_{1}\right)\right\}dy$.

### Univariate CRPS derivation

The CRPS function for genomic selection can be expressed as $CRPS\left(F_{Y_{o}},\mu_{s}\right)=E_{F}\left|Y_{o}-\mu_{S}\right|-\frac{1}{2}E_{F}|Y_{o}-Y_{o}^{’}|$. If we assume that $F_{Y_{o}}\left(y_{o}\right)=N(\mu_{2},\sigma^{2})$ and use some known results of the folded normal distribution, we can derive an analytic expression of the CRPS function.

Assume that $F_{Y}\left(y\right)=N(\mu ,\sigma^{2})$, where μ∈ℝ, and σ>0. Let W=|Y|, then $F_{W}\left(w\right)=N^{f}(\mu ,\sigma^{2})$, where $N^{f}$ means a folded normal distribution. Its probability density function is

$$\begin{array}{l} f\left(w\right)=\frac{1}{\sigma }\left[\varphi \left(\frac{w-\mu }{\sigma }\right)+\varphi \left(\frac{w+\mu }{\sigma }\right)\right]\\ =\frac{1}{\sigma \sqrt{2\pi }}\left\{\exp\left(-\frac{1}{2}{\left(\frac{w+\mu }{\sigma }\right)}^{2}\right)+\exp\left(-\frac{1}{2}{\left(\frac{w-\mu }{\sigma }\right)}^{2}\right)\right\},\;\;\;\;\;w\in \left[0,\infty \right). \end{array}$$

The expectation of W is

$$E\left(W\right)=\mu \left[1-2\Phi (-\mu /\sigma )\right]+\sigma \sqrt{\frac{2}{\pi }}\exp\left(-\mu^{2}/2\sigma^{2}\right).$$

Details of folded normal distribution can be found in Tsagris *et al.* (2014). Now, we will proceed to evaluate $CRPS\left(F_{Y_{o}},\mu_{S}\right)=E_{F}\left|Y_{o}-\mu_{S}\right|-\frac{1}{2}E_{F}|Y_{o}-Y_{o}^{’}|$ as follows. Note that $F_{\left|Y_{o}-Y_{o}^{’}\right|}(\cdot )=N^{f}(0,\sigma^{2})$ and $F_{\left|Y_{o}-\mu_{S}\right|}\left(\cdot \right)=N^{f}\left(\mu_{2}-\mu_{s},\sigma^{2}\right)$. Then, using the previous results, we have that

$$\begin{array}{l} E\left|Y_{o}-{Y^{\prime }}_{o}\right|=\frac{2\sigma }{\sqrt{\pi }}\;,\\ E\left|Y_{o}-\mu_{s}\right|=\left(\mu_{2}-\mu_{s}\right)\left[1-2\Phi \left(-\frac{\mu_{2}-\mu_{s}}{\sigma }\right)\right]+\sigma \sqrt{2/\pi }\exp\left(-\frac{1}{2\sigma^{2}}{\left(\mu_{2}-\mu_{s}\right)}^{2}\right)\\ =\left(\mu_{2}-\mu_{s}\right)\left[1-2\Phi \left(\frac{\mu_{s}-\mu_{2}}{\sigma }\right)\right]+2\sigma \frac{1}{\sqrt{2\pi }}\exp\left(-\frac{1}{2}{\left(\frac{\mu_{2}-\mu_{s}}{\sigma }\right)}^{2}\right)\\ =\left(\mu_{2}-\mu_{s}\right)\left[1-2\Phi \left(\frac{\mu_{s}-\mu_{2}}{\sigma }\right)\right]+2\sigma \varphi \left(\frac{\mu_{2}-\mu_{s}}{\sigma }\right). \end{array}$$

Simplifying,

$$\begin{array}{l} CRPS\left(F_{Y_{o}},\mu_{s}\right)=E_{F}\left|Y_{o}-\mu_{S}\right|-\frac{1}{2}E_{F}\left|Y_{o}-{Y^{\prime }}_{o}\right|\\ =\left(\mu_{2}-\mu_{s}\right)\left[1-2\Phi \left(\frac{\mu_{s}-\mu_{2}}{\sigma }\right)\right]+2\sigma \varphi \left(\frac{\mu_{2}-\mu_{s}}{\sigma }\right)-\frac{1}{2}\frac{2\sigma }{\sqrt{\pi }}\\ =\sigma \left[\frac{\mu_{s}-\mu_{2}}{\sigma }\left(2\Phi \left(\frac{\mu_{s}-\mu_{2}}{\sigma }\right)-1\right)+2\varphi \left(\frac{\mu_{s}-\mu_{2}}{\sigma }\right)-\frac{1}{\sqrt{\pi }}\right]\\ =-\sigma \left[\frac{1}{\sqrt{\pi }}-2\varphi \left(\frac{\mu_{s}-\mu_{2}}{\sigma }\right)-\left(\frac{\mu_{s}-\mu_{2}}{\sigma }\right)\left(2\Phi \left(\frac{\mu_{s}-\mu_{2}}{\sigma }\right)-1\right)\right]. \end{array}$$

### R Code for Univariate Loss Functions

The proposed univariate loss functions use MCMC samples from posterior distributions after a burn-in time period of parameters described in the paper obtained from the BGLR R package.

Inputs of loss function in R language:

• Xb: matrix of MCMC samples of the mean of the candidates of the unobserved phenotype values. Rows for lines, columns for MCMC samples ($\mu_{2}$).

• MU: vector of MCMC samples of the population mean ($\mu_{1}$).

• sigma: vector of MCMC samples of the population standard deviation ($\sigma_{1}$).

• y_c: truncation point (scalar).

• Nsel: number of the candidates to be selected.

• alpha: value indicating the proportion of selected individuals from the candidate set.

Output:

• id of selected lines with the minimum posterior expected losses.

**Table:**

Selection through Standard Method (Std)
selStd <- function(Xb, y_c, Nsel, MU, sigma) {
yHat <- apply(Xb, 1, mean, na.rm = TRUE)
selected <- order(yHat, decreasing = TRUE)[1:Nsel]
return(selected)
}

Selection through Kullback-Leibler (KL)
selKL <- function(Xb, y_c, Nsel, MU, sigma) {
n.mcmc <- ncol(Xb)
n.lines <- nrow(Xb)
losses <- matrix(0, n.lines, n.mcmc)
for(i in 1:n.mcmc) {
mu1 <- MU[i]
mu2 <- Xb[,i]
sd <- sigma[i]
yz <- (y_c - mu1) / sd # standardizing
Z <- 1 - pnorm(yz) # normalization factor
dens <- dnorm(yz, mean = 0, sd = 1) # density
prob <- pnorm(yz, mean = 0, sd = 1) # probability
muT <- mu1 + sd * (dens / (1 - prob))
# posterior predictive function at each iter.
yppdf <- sapply(mu2, function(ypred) {
rnorm(1, mean = ypred, sd = sd)})
losses[,i] <- -log(Z) + ((muT - yppdf)^2 –
(muT - mu1)^2) / (2 * sd^2)
}
e.loss <- apply(losses, 1, mean, na.rm = TRUE)
selected <- order(e.loss, decreasing = FALSE)[1:Nsel]
return(selected)
}

Selection through Continuous Ranked Probability Score (CRPS)
selCRPS <- function(Xb, y_c, Nsel, MU, sigma) {
n.mcmc <- ncol(Xb)
n.lines <- nrow(Xb)
losses <- matrix(0, n.lines, n.mcmc)
for(i in 1:n.mcmc){
mu1 <- MU[i]
mu2 <- Xb[,i]
sd <- sigma[i]
zz <- (y_c-mu1)/sd
muT <- mu1 + sd*(dnorm(zz)/(1-pnorm(zz)))
# posterior predictive function at each iter.
yppdf <- sapply(mu2, function(ypred) {
rnorm(1, mean = ypred, sd = sd)})
yz <- (muT-yppdf) / sd # standardizing
dens <- dnorm(yz, mean = 0, sd = 1) # density
prob <- pnorm(yz, mean = 0, sd = 1) # probability
losses[,i] <- -1 * (sd * (1 / sqrt(pi) –
2 * dens - yz * (2 * prob - 1)))
}
e.loss <- apply(losses, 1, mean, na.rm = TRUE)
selected <- order(e.loss, decreasing = FALSE)[1:Nsel]
return(selected)
}

Selection through Linear-Linear (Lin-Lin)
selLin <- function(Xb, y_c, Nsel, MU, sigma, alpha) {
n.mcmc <- ncol(Xb)
n.lines <- nrow(Xb)
losses <- matrix(0, n.lines, n.mcmc)
for(i in 1:n.mcmc){
mu1 <- MU[i]
mu2 <- Xb[,i]
sd <- sigma[i]
z = (y_c-mu1)/sd
prob <- 1-pnorm(z)
dens <- dnorm(z)
bias <- sd*dens/prob
muT <- mu1 + bias
# posterior predictive function at each iter.
yppdf <- sapply(mu2, function(ypred) {
rnorm(1, mean = ypred, sd = sd)})
losses[,i] <- ((alpha-ifelse(yppdf < muT, 1, 0))*(yppdf-muT))
}
e.loss <- apply(losses, 1, mean, na.rm = TRUE)
selected <- order(e.loss, decreasing = FALSE)[1:Nsel]
return(selected)
}

### R code for Multivariate Loss Function

The multivariate loss functions proposed were adapted to use MCMC samples from posterior distributions of parameters after a burn-in time period obtained from the MTM R package.

Inputs of loss function in R language:

• Xb: matrix of MCMC samples of the mean of the candidates of unobserved phenotype values. In columns are the lines, in rows are M MCMC samples ($\mu_{2}$).

• MU: matrix of MCMC samples of population mean, in columns are the means for each trait, in rows MCMC samples ($\mu_{1}$).

• K: matrix of MCMC samples of population var-covariance, in columns each component of covariance matrix, in rows MCMC samples (K).

• y_c: vector of truncation points for each trait, length equal to the number of traits.

• Nsel: number of the candidates to be selected.

• tau: vector of length equal to number of traits. Each value of the vector indicates the proportion of selected individuals from the candidate set.

Output:

• id of selected lines with the minimums posterior expected losses.

**Table:**

Selection through Multivariate Kullback-Leibler (KL)
selKL <- function(Xb, MU, K, y_c, Nsel) {
Xb <- as.matrix(Xb); MU <- as.matrix(MU); K <- as.matrix(K);
n.traits <- ncol(MU)
n.lines <- ncol(Xb)/n.traits
n.mcmc <- nrow(Xb)
losses <- matrix(0, nrow = n.lines, ncol = n.mcmc)
Kall <- alply(K, 1, function(V) as.matrix(nearPD(xpnd(unlist(V)))$mat))
Kallinv <- llply(Kall, solve)
Xb <- apply(Xb, 1, split, f = rep(1:n.traits, each = n.lines))
for(i in 1:n.mcmc) {
mu1 <- as.vector(MU[i,])
mu2 <- do.call(cbind, Xb[[i]])
K <- Kall[[i]]
Kinv <- Kallinv[[i]]
muS <- as.vector(mtmvnorm(mean = mu1, sigma = K, lower = y_c,
upper = rep(Inf, length(y_c)),
doComputeVariance = FALSE)$tmean)
Z = pmvnorm(lower = y_c, upper = Inf, mean = mu1, sigma = K)
# posterior predictive distribution at each iteration.
yppdf <- as.matrix(t(apply(mu2, 1, function(ypred) {
rmvnorm(1, mean = ypred, sigma = K)})))
S <- muS-mu1 # selection differential
UKU <- as.numeric(t(S)%*%Kinv%*%S)
muSmu2 <- sweep(yppdf,2,muS,’-’) # muS - mu2
losses[,i] <- as.vector(apply(muSmu2, 1, function(x) {
0.5*(t(x)%*%Kinv%*%x-UKU)-log(Z) }))
}
e.loss <- apply(losses, 1, mean, na.rm = TRUE)
selected <- order(e.loss, decreasing = FALSE)[1:Nsel]
return(selected)
}

Selection through Energy Score (EnergyS)
selEnergyS <- function(Xb, MU, K, y_c, Nsel) {
Xb <- as.matrix(Xb); MU <- as.matrix(MU); K <- as.matrix(K);
n.traits <- ncol(MU)
n.lines <- ncol(Xb)/n.traits
n.mcmc <- nrow(Xb)
losses <- matrix(0, nrow = n.lines, ncol = n.mcmc)
Kall <- alply(K, 1, function(V) as.matrix(nearPD(xpnd(unlist(V)))$mat))
Xb <- apply(Xb, 1, split, f = rep(1:n.traits, each = n.lines))
for(i in 1:n.mcmc) {
mu1 <- as.vector(MU[i,])
mu2 <- do.call(cbind, Xb[[i]])
K <- Kall[[i]]
muS <- as.vector(mtmvnorm(mean = mu1, sigma = K, lower = y_c,
upper = rep(Inf, length(y_c)),
doComputeVariance = FALSE)$tmean)
# posterior predictive distribution at each iteration.
yppdf <- as.matrix(t(apply(mu2, 1, function(ypred) {
rmvnorm(1, mean = ypred, sigma = K)})))
yppdf_*P* < - as.matrix(t(apply(mu2, 1, function(ypred) {
rmvnorm(1, mean = ypred, sigma = K)})))
muSmu2 <- sweep(yppdf,2,muS,’-’) # X-y
mu2mu2 <- yppdf-yppdf_p # X-X’
xj_y <- as.vector(apply(muSmu2, 1, function(x) sqrt(sum(x^2))))
xj_xjp <- as.vector(apply(mu2mu2,1,function(x) 0.5*sqrt(sum(x^2))))
losses[,i] <- xj_y - xj_xjp
}
e.loss <- apply(losses, 1, mean, na.rm = TRUE)
selected <- order(e.loss, decreasing = FALSE)[1:Nsel]
return(selected)
}

Selection through Multivariate Asymmetric Loss Function (MALF)
selMALF <- function(Xb, MU, K, y_c, Nsel, tau) {
Xb <- as.matrix(Xb); MU <- as.matrix(MU); K <- as.matrix(K);
n.traits <- ncol(MU)
n.lines <- ncol(Xb)/n.traits
n.mcmc <- nrow(Xb)
losses <- matrix(0, nrow = n.lines, ncol = n.mcmc)
Kall <- alply(K, 1, function(V) as.matrix(nearPD(xpnd(unlist(V)))$mat))
Xb <- apply(Xb, 1, split, f = rep(1:n.traits, each = n.lines))
for(i in 1:n.mcmc) {
mu1 <- as.vector(MU[i,])
mu2 <- do.call(cbind, Xb[[i]])
K <- Kall[[i]]
muS <- as.vector(mtmvnorm(mean = mu1, sigma = K, lower = y_c,
upper = rep(Inf, length(y_c)),
doComputeVariance = FALSE)$tmean)
# posterior predictive distribution at each iteration.
yppdf <- as.matrix(t(apply(mu2, 1, function(ypred) {
rmvnorm(1, mean = ypred, sigma = K)})))
error <- -1*sweep(yppdf,2,muS,’-’) # mu2 - muS
abs.error <- abs(error)
sum.e <- rowSums(abs.error)
score <- sum.e + t(error %*% tau)
losses[,i] <- score
}
e.loss <- apply(losses, 1, mean, na.rm = TRUE)
selected <- order(e.loss, decreasing = FALSE)[1:Nsel]
return(selected)
}
